# Lifestyle Intervention for Cardiovascular Disease Risk Factors in Jeddah, Saudi Arabia

**DOI:** 10.7759/cureus.11791

**Published:** 2020-11-30

**Authors:** Jumana H Khouja, Badr Al Jasir, Amina A Bargawi, Mohammed Kutbi

**Affiliations:** 1 Preventive Medicine, King Abdullah International Medical Research Center, Jeddah, SAU; 2 Preventive Medicine, King Saud Bin Abdulaziz University for Health Sciences, Jeddah, SAU

**Keywords:** cardiovascular diseases, framingham risk score, life style modification, randomized controlled trial

## Abstract

Background

Cardiovascular disease (CVD) remains the major cause of global mortality. Applying a comprehensive interventional program may reduce the incidence of cardiovascular disease and its complications.

Objective

This study compared the effects of a three-month intervention involving lifestyle modification and physical activity with standard care in women ≥30 years having a moderate to high risk of CVD, with respect to improving physical activity and cardiovascular disease risk factors at the National Guard Residential City in Jeddah, Saudi Arabia, in 2015.

Methods

The effects of this community-based lifestyle program were assessed through a randomized controlled trial from January 1st to September 6th, 2015. Women in the intervention group (n = 31) received health education, exercise training, and diet counselling as individuals and in groups according to the participant’s risk. Women in the control group (n = 28) received one health education session at the screening site. The primary outcome was the proportion of women with moderate Framingham risk scores (FRS) reducing their risk by 10% and the proportion of women with high FRS reducing their risk by 25%. The secondary outcome was the proportion of women reducing their risk by ≥1 risk category.

Results

The mean participant age was 42 ± 8 years. At three-month’s follow-up, reductions were greater in the intervention group and the difference between groups was statistically significant (p < 0.05). Lifestyle intervention program significantly reduced systolic blood pressure (-9.2 mmHg), blood glucose (-45 mg/dL) and Framingham risk score (-13.6). Linear regression analysis revealed a significant improvement in the Framingham risk score (p < 0.01).

Conclusion

In a population of women with moderate-to-high risk of CVD, a personalized lifestyle modification program showed positive association in improving the 10-year cardiovascular Framingham risk score after three months.

## Introduction

Cardiovascular disease (CVD) includes various conditions such as hypertension, coronary heart disease (CHD), myocardial infarction, angina pectoris, heart failure, and stroke [1]. Although CVD incidence and mortality have markedly declined in recent decades [2], CVD remains the leading cause of death worldwide [3]. Women tend to have CVD in the more developed areas of the world. CVD accounted for approximately 0.3 million deaths in 2009 in the United States, and was responsible for one in every four female deaths [4]. Almost two-thirds (64%) of women who die suddenly of CHD have had no previous symptoms [5]. According to the World Heart Federation, CVD is the most serious neglected health problem affecting women worldwide [6], being responsible for 17.3 million deaths in 2008 (30% of all mortalities) [7]. According to the World Health Organization statistics for 2008, most of the mortalities in Saudi Arabia were due to non-communicable chronic diseases. Of the 413 deaths per 100,000 individuals in 2009, 144 (35%) were due to CVD [7]. CVD is one of the main health problems in Saudi Arabia, representing the third most common cause of hospital-based mortality after accidents and senility [8]. Prevalence of modifiable risk factors in Saudi women that have been clinically proven to influence cardiovascular health include diabetes (9.6% to 27.6%), high blood pressure (21.8%), hypercholesterolemia (24.5%), being overweight (27%) or obese (40.23%), insufficient physical activity (less than 30 minutes of moderate activity 5 times/week or 20 min of vigorous activity 3 times/week, or the equivalent) (53.2% to 98.1%), unhealthy diet, and smoking (1.1% to 9.1%). Other non-modifiable risk factors include age, gender, family history, and race or ethnicity [9].

Many interventions have been developed to prevent and treat CVD. Cardiovascular disease risk factors can act synergistically to increase the risk of developing heart disease; therefore, interventions that focus on modifying multiple risk factors may be more effective in reducing risk than those that focus on only one risk factor at a time. A multidisciplinary approach combining diet, exercise, and behavioral change can improve survival, prevent or reduce recurrent events and procedures, and improve quality of life [1]. According to the Centers for Disease Control and Prevention, six of 10 deaths from CVD are preventable [10]. Therefore, it is important to develop more comprehensive approaches for the primary prevention of CVD [6]. The public health burden of a sedentary lifestyle necessitates interventions that can reach enough women to make a health impact on a wide scale [11]. In addition, the Saudi female’s social culture promotes poor nutritional habits and limits physical activity, increasing the risk of CVD. Applying a comprehensive interventional program according to the individual’s risk factors may decrease the prevalence of CVD and its associated complications, and reduce the burden on the healthcare system. However, this concept has not been sufficiently investigated in Saudi Arabia. This study aimed to assess the effects of a personalized lifestyle modification program targeting women ≥30 years at moderate-to-high risk of CVD. (PDF: Khouja J, Al Jasir B, Bargawi A, Kutbi M. Lifestyle Intervention for Cardiovascular Disease Risk Factors among Female Residents of the National Guard Residential City, Jeddah, Saudi Arabia: A Randomized Controlled Trial; 2017), https://clinicaltrials.gov/ProvidedDocs/15/NCT03532815/Prot_001.pdf

## Materials and methods

The study was conducted in the National Guard Residential City, Jeddah, which comprises five geographical sections containing 1229 villas each. A randomized controlled trial was conducted from January 1st to September 6th, 2015. All female residents of the National Guard Residential City aged ≥30 years were screened and their Framingham risk scores (FRSs) were calculated. To be included in the study, the participants had to be at moderate-to-high risk of CVD according to FRS. Those with a low CVD risk according to FRS, who were pregnant, or diagnosed with CVD were all excluded.

The sample size was calculated using the formula for superiority trials:

n = (Zα + Zβ)2 × (p1 × (1 − p1) + p2 (1 − p2)/(p2 − p1)2

(Where n is the sample size, α is the probability of type I error = 5%, β is the probability of type II error = 20%, p1 is the expected success proportions of sample one and p2 is the expected success proportions of sample two).

**Figure 1:**
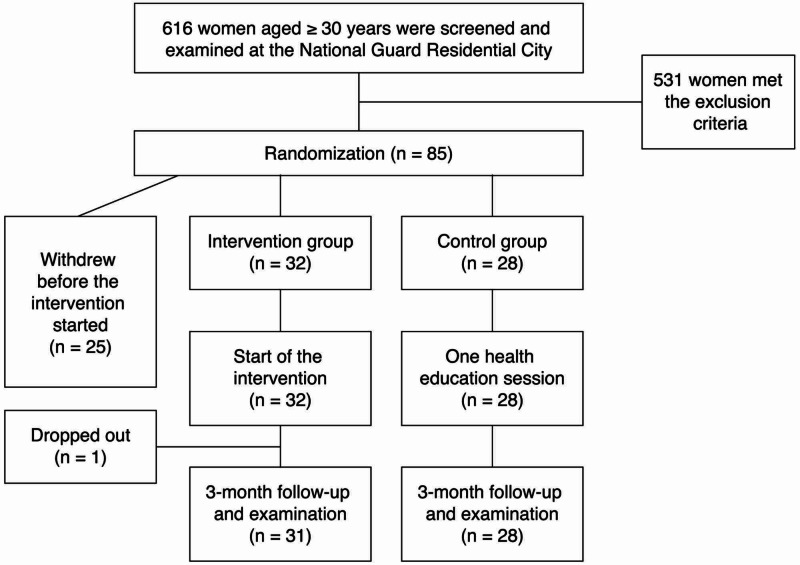
Flowchart from recruitment to completion of the follow-up of the participants at the National Guard Residential City in Jeddah, Saudi Arabia, in 2015

The sample of screened women was 616 with a response rate of 50%. From the total sample, 85 (13.8%) participants were in the moderate- and high-risk groups. The intervention arm included 42 participants; and the control arm, 43 participants. Eligible participants randomly chose a card that assigned them to the intervention or control group. The investigator was blinded to the participants’ group assignment. The participants assigned to the intervention arm of the study underwent a lifestyle modification program, including exercise training sessions and diet counselling. Those assigned to the control arm received one session of health education at the screening site (Figure 1). At the start of the study, all the households in the residential city were visited and informed about the study by a group of trained research assistants supported by the required documents.

Women received basic education about CVD and its associated risk factors. The participants were interviewed and screened by the investigator and two trained nurses, using the validated and widely used Coronary Risk Profile (CRP) questionnaire from Wellsource Inc. (Portland, OR, USA) [12]. The CRP questionnaire was translated to Arabic and tested for content and face validity to ensure appropriate clarity and fulfillment of the study objectives. Three experts reviewed the final version of the questionnaire and pilot testing was conducted among a group of 10 women. The results were reviewed and modifications were applied.

The questionnaire collects data on health history, smoking habits, physical activity, eating practices, and social factors. Data from the CRP questionnaire were used to calculate individualized FRS. The risk estimate and factors that influenced cardiovascular disease risk were explained for each individual.

Eligible participants were recruited in April 2015 and received the intervention in May 2015. The three-month follow-up was completed at the beginning of September 2015.

Clinical measurements

The participants’ weight was measured without shoes and the height was measured using a stadiometer. Body mass index (BMI) was calculated as weight in kilograms divided by height in meters squared. Waist circumference was measured in centimetres from the point midway between the inferior margin of the last rib and the iliac crest. Blood pressure was assessed in the sitting position using a standard mercury sphygmomanometer. A fasting blood sample was obtained using the CardioChek PA System (Polymer Technology Systems, Inc., Indianapolis, IN, USA) which provides results on cholesterol, high-density lipoprotein (HDL), glucose, and triglyceride levels. The results met the accuracy guidelines established by the National Cholesterol Education Program of the National Institutes of Health [12].

Blood samples were obtained at baseline and at three months. According to the above-mentioned measures, FRS was calculated to estimate the 10-year risk of a major cardiovascular event and for risk categorization into low (<10% FRS), moderate (10%-19% FRS), or high risk (≥20% FRS).

Those with moderate and high risks were evaluated for their metabolic syndrome status. They were classified as having metabolic syndrome if they satisfied three of the five following criteria (based on the criteria established by the National Heart, Lung and Blood Institute for the diagnosis of metabolic syndrome) [13,14]: (1) abdominal obesity, determined by increased waist circumference >88 cm in women; (2) increased triglyceride levels ≥1.7 mmol/L (150.6 mg/dL); (3) reduced HDL level of <1.30 mmol/L (50.2 mg/dL); (4) elevated blood pressure ≥130 mmHg systolic or ≥85 mmHg diastolic blood pressure; and (5) increased fasting glucose level ≥6.1 mmol/L (109.8 mg/dL). Individuals with a high fasting blood glucose level ≥126 mg/dL (7.0 mmol/L), high blood pressure ≥140/90 mmHg, or total cholesterol level ≥240 mg/dL (6.2 mmol/L) were referred to the primary health-care center for further assessment by their respective physicians. Nevertheless, they still participated in the study.

Study intervention

The participants in the intervention arm of the study received a program of lifestyle modification, including health education, exercise training sessions, and diet counselling delivered by the researcher, health educator, physiotherapist, and nutritionist. Anthropometric measures, blood pressure, blood glucose level, total cholesterol level, triglyceride level, and HDL level were measured at baseline and after three months. Those assigned to the control arm received one session of health education at the screening site and underwent the same measurements. The intervention was a one-month program based on the ‘Be Active your way’ developed by the United States Department of Health and Human Services and the National Guidelines for Management of Cardio-metabolic Risk Factors, developed by the Saudi Ministry of Health [15,16].

The intervention was conducted in the morning and afternoon at the officer’s sports club in the residential city. The initial visit included assessment of the participant’s cardiovascular disease risk. The intervention was conducted in group and individualized sessions according to the participants’ needs and risk status. The lifestyle intervention program was designed to teach women how to incorporate a daily 30-minute physical activity of moderate-intensity into their routine and how to select and eat nutritious foods. The intervention arm included 31 participants and the intervention’s duration for the whole group was four weeks. About 10 individuals were included daily. Each participant had two visits to undertake the intervention over the period of four weeks (once every other week).

During each visit, a two-hour face-to-face educational session was conducted. During the two hours, each participant had to rotate through all the sections (researcher, health educator, exercise specialist, and nutritionist) and received different educational materials from trained staff. Various motivational measures were considered to keep meetings interesting and to boost attendance, such as contests and individual rewards.

After the active intervention period, the participants were encouraged monthly by phone to maintain at least the 30 minutes of physical activity daily, a healthy diet, and to monitor the effect of these modifications on their health.

The participants in the control (n = 28) arm of the study received standard care at their primary health-care centers and one health education session of 30 minutes about CVD and its risk factors. During this session, they were given information about the prevention of these diseases by adopting healthy behaviors, including diet and exercise.

Data analysis

Data entry and statistical analysis were conducted using the Statistical Package for Social Sciences version 22 (IBM Corp., Armonk, NY, USA). Demographic and clinical data were analyzed using descriptive statistics, including means, standard deviations, and frequencies. Pre- and post-outcome measures were compared using chi-square and t-test. Repeated measures analysis of variance was used to compare the three-month effect between the intervention and control group. The effects of the intervention on multiple outcomes were evaluated using multiple linear regression techniques. Linear regression analysis was used for FRS difference. This research used an alpha level of 0.05 as the threshold for statistical significance.

Limitations of the study

The follow-up duration could have been longer to examine the long-term effects of the intervention and of participants’ adherence to any acquired lifestyle changes. Another limitation is the generalizability of the results, the small sample size from a group of women in the NG residential city in Jeddah might not be generalizable to the rest of the country. However, since most women in Saudi have similar risk factors the results from this study shed a light on a possible intervention scheme that should be studied in larger groups in the country.

## Results

The mean age of the intervention and control group was 49 ± 6.5 and 48 ± 5.6 years, respectively. The proportion of the employed among the participants in the intervention group (39%) was significantly greater than that in the control group (11%) (p = 0.014). Other demographic details are shown in Table 1.

**Table 1:** Sociodemographic characteristics of the participants (n = 59) at the National Guard Residential City in Jeddah, Saudi Arabia, 2015 Data are presented as number and percentage (%). * Statistically significant at p < 0.05.

Socio-demographic characteristic	Intervention group (n = 31)	Control group (n = 28)	p-value
Age in years			
Mean ± SD	49 ± 6.5	48 ± 5.6	0.315
30-39	1 (3%)	0 (0%)	
40-49	16 (52%)	19 (68%)	
50-59	12 (39%)	9 (32%)	
60-69	2 (7%)	0 (0%)	
Marital status			
Married	31 (100%)	26 (93%)	0.318
Divorced	0	1 (3.5%)	
Widow	0	1 (3.5%)	
Education			
Illiterate	7 (22%)	16 (57%)	0.065
Read/write	3 (10%)	2 (7%)	
Primary	3 (10%)	3 (11%)	
Intermediate	4 (13%)	2 (7%)	
High school	5 (16%)	0 (0%)	
University	9 (29%)	5 (18%)	
Occupation			
Housewife	19 (61%)	25 (89%)	0.014*
Employee	12 (39%)	3 (11%)	
Income/month (SR)			
<5000	4 (13%)	9 (32%)	0.031*
5000-10000	12 (39%)	14 (50%)	
>10000	15 (48%)	5 (18%)	

Data on dietary habits and physical activity showed that 62% of participants did not regularly exercise. Approximately 38% habitually missed breakfast. Once-a-day fruit and vegetable intake were satisfactory (42% and 46%, respectively).

As shown in Table 2, five parameters significantly changed with the application of the intervention. BMI decreased from 33.7 ± 6.6 kg/m^2^ to 32.9 ± 6.5 kg/m^2^ (p < 0.01). HDL level increased from 40.6 ± 17.4 mg/dL to 46.5 ± 8.8 mg/dL (p = 0.04). Systolic blood pressure decreased from 141 ± 18.4 mmHg to 127.6 ± 13.9 mmHg (p < 0.01), and diastolic blood pressure decreased from 91.9 ± 11.5 mmHg to 87.42 ± 9.7 mmHg (p = 0.03). In addition, FRS significantly changed, decreasing from 13.02 ± 3.3 to 8.1 ± 5.03 (p < 0.01).

**Table 2:** Changes in modifiable risk factors among the intervention (n = 31) and control group (n = 28) at three-month follow-up compared to baseline at the National Guard Residential City in Jeddah, Saudi Arabia, 2015 * Statistically significant at p < 0.05. BMI: Body mass index, HDL: High-density lipoprotein; LDL: Low-density lipoprotein, BP: Blood pressure, FRS: Framingham risk score.

Risk factor	Control Group				Intervention Group			
	Mean ± SD		Mean difference	p-value		Mean ± SD	Mean difference	p-value
	Preintervention	Postintervention			Preintervention	Postintervention		
BMI (kg/m^2^)	30.8 ± 8	30.6 ± 8	-0.18	0.08	33.7 ± 6.6	32.9 ± 6.5	-0.83	0.01*
Waist circumference (cm)	105.8 ± 12	105.6 ± 11	-0.14	0.86	102 ± 13	100 ± 22	1.87	0.58
Total cholesterol (mg/dL)	176.6 ± 37	184.2 ± 39	4.62	0.53	190 ± 51	186 ± 48	3.77	0.76
HDL (mg/dL)	47 ± 12	44 ± 11	-1.19	0.67	40.6 ± 17.4	46.5 ± 8.8	5.96	0.04*
LDL (mg/dL)	107 ± 42.7	122.8 ± 45.9	15.82	0.04*	115 ± 42	104 ± 36	11.41	0.15
Triglycerides (mg/dL)	161 ± 84	137 ± 52	-23.67	0.15	171 ± 103	169 ± 84	-1.35	0.93
Systolic BP (mmHg)	138 ± 12	136 ± 12	-1.71	0.41	141 ± 18.4	127.6 ± 13.9	-13.35	0.01*
Diastolic BP (mmHg)	77.8 ± 8	77.6 ± 10	0.21	0.86	91.9 ± 11.5	87.42 ± 9.7	-4.51	0.03*
Fasting blood glucose (mg/dL)	152 ± 100	142 ± 66	-10.40	0.53	104 ± 47	97 ± 45	-6.66	0.47
FRS	18.6 ± 7.5	21.7 ± 8.5	3.04	0.04*	13.02 ± 3.3	8.1 ± 5.03	-4.93	0.01*

The chi-square correlation test revealed a significant difference (p < 0.01) in Framingham risk category level (low, moderate, and high) in the intervention and control group before and after the intervention (Table 3). In the intervention group, 22 women (71%) shifted from the moderate to low level, seven (23%) remained at the moderate level, and one (3%) remained at the high level. In addition, one woman (3%) shifted from the high to moderate level.

**Table 3:** Post-pre shift in Framingham risk category in the intervention and control group (n = 59) at three months in the National Guard Residential City, Jeddah, Saudi Arabia, 2015

		Risk category-post			p-value
		Low	Moderate	High	
Risk category-pre (control)	Moderate	2	10	7	0.03
	High	0	1	8	
Risk category-pre (intervention)	Moderate	22	7	0	0.01
	High	0	1	1	

Table 4 illustrates the effect of the intervention after three months. Significant differences were noted in blood pressure (p = 0.09) and blood glucose level (p = 0.03). However, no significant difference was observed in blood lipids levels, BMI, and waist circumference. FRS differed significantly (p < 0.01) between the intervention and control groups. Table 4 shows that metabolic syndrome status did not differ significantly between the intervention and control groups after three months of the intervention.

**Table 4:** The three-month effect of the lifestyle modification program on the intervention (n = 31) and control (n = 28) groups, with respect to the metabolic syndrome (n = 59) at the National Guard Residential City in Jeddah, Saudi Arabia, 2015 * Statistically significant at p < 0.05. BMI: Body mass index, HDL: High-density lipoprotein; LDL: Low-density lipoprotein, BP: Blood pressure, FRS: Framingham risk score.

Variable		Mean	SD	95% Confidence Interval for Mean		p-value
				Lower bound	Upper bound	
Total Cholesterol (mg/dL)	Control group	190.71	44.50	173.46	207.97	1.00
	Intervention group	190.71	51.43	171.84	209.58	
BMI (kg/m^2^)	Control group	30.62	8.13	27.47	33.78	0.23
	Intervention group	32.91	6.52	30.52	35.30	
HDL (mg/dL)	Control group	43.93	11.56	39.44	48.41	0.32
	Intervention group	46.55	8.82	43.31	49.78	
Triglycerides (mg/dL)	Control group	137.36	52.42	117.03	157.69	0.08
	Intervention group	169.84	84.88	138.70	200.98	
LDL (mg/dL)	Control group	122.79	45.86	105.00	140.57	0.53
	Intervention group	115.61	42.94	99.86	131.36	
Waist circumference (cm)	Control group	105.68	11.96	101.04	110.32	0.27
	Intervention group	102.03	13.14	97.21	106.85	
Systolic BP (mmHg)	Control group	136.93	12.33	132.15	141.71	0.09*
	Intervention group	127.65	13.95	122.53	132.76	
Blood glucose (mg/dL)	Control group	142.36	66.21	116.68	168.03	0.03*
	Intervention group	97.61	44.36	81.34	113.89	
FRS	Control group	21.67	8.46	18.39	24.95	0.01*
	Intervention group	8.09	5.03	6.24	9.93	

Finally, a linear regression revealed that the intervention showed a statistically significant improvement in FRS between the two groups (p < 0.01), when all other co-variates were adjusted for. However, being married was a negative predictor of the cardiovascular risk difference (Table 5).

**Table 5:** Linear regression model for the Framingham risk score difference at three-month adjusting for demographics, social factors and co-morbid conditions, only significant results are displayed (n = 59) at the National Guard Residential City in Jeddah, Saudi Arabia, 2015

Coefficients						
Model		Unstandardized Coefficients		Standardized Coefficients	t	p-value
		B	SE	Beta		
	(Constant)	1.41	2.04		0.69	0.49
	Intervention	8.36	1.62	0.60	5.16	0.01
	Married	-6.58	2.38	-0.30	-2.76	0.08
	40-49 years	3.36	1.63	0.22	2.05	0.04
	R^2^: 0.43					
	Adjusted R^2^: 0.40					

## Discussion

A randomized controlled trial was conducted to assess the effect of a community-based lifestyle modification program in women with a moderate-to-high risk of CVD.

Despite the short duration of the study, results demonstrate that this multidisciplinary intervention, tailored to the individual’s risk, showed promising results in reducing some of the CVD risk factors and the overall FRS, compared to standard care in women at moderate-to-high risk of CVD after three months. The current study showed significant favorable changes in some of the cardiac risk factors in the intervention group. BMI reduced by 0.8 kg/m^2^ and HDL improved by 5.9 mg/dL. In addition there were improvements in systolic and diastolic blood pressure by 13.3 and 4.5 mmHg, respectively. FRS also decreased by 4.9. This agrees with previous Canadian and American studies, which showed improvements in the same cardiac risk factors [3].

The study also revealed non-favorable changes in some of the cardiac risk factors in the control group. Low-density lipoprotein (LDL) worsened by 15.8 mg/dL and FRS increased by 3.04. Other parameters did not differ significantly. The intervention by the multidisciplinary team could have motivated participants in the intervention group to take better charge of their health habits compared to the controls. This explanation is in congruence with other randomized controlled trials showing the effect of lifestyle intervention on CVD risk [2, 17].

At three-month follow-up, the intervention group had significantly lower systolic blood pressure and blood glucose levels compared to the control. This result is in accordance with findings from other lifestyle intervention studies [2,3,11]. The intervention and control group also differed regarding blood glucose, which was lower in the intervention group by 45 mg/dL, as seen in the ANCHOR study [3].

Although there was an improvement in HDL levels in the intervention group and the LDL level worsened in the control group, the intervention did not produce any significant difference in the overall blood lipid profile between both groups. These results compare favorably with the lifestyle intervention studies conducted by Eriksson [2], Pazoki et al. [11], and Ebrahim et al. [18]. BMI decreased in the intervention group, but overall, reductions in BMI and waist circumference were not significant. The same findings were noted in another community-based healthy heart program study conducted in Iran [11].

Rates of metabolic syndrome did not differ significantly between the intervention and control groups; however, other pre-post interventional studies showed an improvement in the status of metabolic syndrome. This could have been achieved through the effect of the behavioral counseling which is the cornerstone approach in the literature [3].

Despite this, a significant reduction in FRS was demonstrated at three months. It is notable that in the intervention group, 71% of women shifted from the moderate to the low risk category and 3% of the participants shifted from the high to moderate risk category, thus, underpinning the intervention’s effectiveness. This improvement was shown in the pre- and post-intervention ANCHOR study [3].

Moreover, FRS differed significantly between the intervention and control groups, decreasing in the intervention group and increasing in the control group. This was expected since most of the modifiable risk factors that are included in the calculation of FRS improved in the intervention group. This result is consistent with another randomized controlled trial on the efficacy of lifestyle intervention in reducing cardiovascular disease risk [17].

The intervention consisting of customized health education, diet counselling and exercise training had a positive effect on the outcome. In addition, the younger age group were more likely to see improved outcomes. This could be due to their enthusiasm to improve their lifestyle and family health in general. However, being married was a negative predictor of the cardiovascular disease risk difference. The responsibilities of marriage might have made them less interested in improving their lifestyle. In agreement with this, some studies which assessed the motives for participating in a lifestyle intervention trial showed that participants were younger, single, had a higher level of education, and were employed [19-20]. Thus, the intervention itself represents a major change for the success of the program and for FRS reduction. These results could be helpful for identifying subjects with greater chances of successful lifestyle intervention in the development of future programs.

## Conclusions

Over the three-month intervention period, the 10-year risk of CVD was successfully reduced among 31 women with moderate-high CVD risk by applying a comprehensive program tailored to the individual’s risk. The overall FRS was improved by the intervention. These results highlight the importance of multifaceted and comprehensive interdisciplinary programs that improve cardiovascular disease risk reduction, encourage healthy behaviors, and promote active lifestyles in persons at risk of CVD.
